# Editorial for the Special Issue: Game Meat and Game Meat Products: Safety, Quality and Consumer Perception

**DOI:** 10.3390/foods11142073

**Published:** 2022-07-12

**Authors:** Raffaella Branciari, David Ranucci

**Affiliations:** Department of Veterinary Medicine, University of Perugia, Via San Costanzo 4, 06126 Perugia, Italy

Consumer interest in game meat has increased in recent years, mainly due to the growing population of some wild species, the “natural sounding” aspect of game meat and its good nutritional value. Consumer choice is mainly influenced by rational motives more than emotional factors, with quality, expressed as nutritional traits linked with healthcare issues [1] as the most important consideration. Different authors recently confirmed the excellent nutritional value, such as high percentage of protein, low fat, proper proportion of n3–n6 polyunsaturated fatty acids and levels of minerals, of numerous game meat species such as Namibian Red Hartebeest (*Alcelaphus buselaphus*) [2], Brown bear (*Ursus arctos*) [3], Hare (*Pedetes capensis*) [4], Red Deer (*Cervus elaphus*) [5], Fallow Deer (*Dama dama*) [6] and Impala (*Aepyceros melampus*) [7]. Moreover, other nutritional factors of game meat should be considered, such as bioactive compounds with antihypertensive properties. In this context, the angiotensin I-converting enzyme (ACE) inhibitory activities are higher in the digested game meats (venison and boar meat) than those of livestock meats (beef and pork) [8]. The commercial potential of game meat also prompted studies on proportions of valuable meat cuts of specific and uncommon wild species, such as Giraffe (*Giraffa camelopardalis angolensis*) [9]. Nonetheless, in many of the species reported, difference in quality traits could be encountered according to age [3], meat cut [2] and season [4]. These factors could be therefore considered during the marketing of game meat concerning the aforementioned species [6]. Sex factors appear to be negligible, especially in species with no sexual dimorphism [7].

Game meat could also be considered for meat preparation and products, in which the peculiar quality traits of the meat could be preserved [10] or even enhanced with a specific ingredient. Taking into consideration that the higher amount of polyunsaturated fatty acid in this meat could negatively affect the oxidative stability of the products, the addition of other fat sources, such as sheep fat in Zebra (*Equus burchelli*) Droëwors [11] or vegetable oils in deer burger [12], should be considered.

Another relevant issue for consumer perception of game meat is its safety. In a study conducted in Poland, almost all consumers believe that the positive impact of game meat is higher than the safety risk, but some search for information and are convinced to counteract the safety risk only by not eating raw meat [13]. Game meat safety issues must be considered separately from livestock animal meat because of the differences in the environment where the animal lives and differences in the first step of the production chain (transport and slaughtering vs. hunting). Hygiene and safety issues could thus be considered according to several factors present in the pre-harvest and hunting phases that are directly pertinent to hunters, such as the duration of storage before skinning, the environmental temperature during hunting and the time between shooting and evisceration, associated with animal weight (considered in wild boar *Sus scropha* and roe deer *Caperolus capreolus*) [14,15]. Thus, in a survey conducted in Italy, the hunter themselves believed that is crucial to improve hunters’ training activities to increase the safety and quality of the final product [16]. If the hunting and post-harvest processes are properly conducted, game meat could follow the same microbiological criteria adopted for slaughtered animals [14]. Under these optimal conditions, the application of decontamination strategies using weak organic acid solution to improve meat shelf-life could be suggested [17]. Indeed, different factors present during hunting could influence either the hygienic level of the meat or its quality traits, affecting its pH, color and other quality characteristics. When game meat of poor quality (e.g., meat with high ultimate pH) is used for meat processing, such as in naturally fermented meat products, microbiological safety risks could occur [18].

The role of hunters must be considered even in terms of the presence of heavy metals in game meat, as Lead (Pb) present in the ammunition used for hunting remains the most significant threat for toxic metals contamination in game meat animals. Monitoring and control of heavy metals in the game meat provided in developing countries must therefore be implemented [19].

## Data Availability

Not Applicable This Special Issue collects researches related to different aspect of Game Meat and Game Meat Products. The contributions here gathered constitute a valuable knowledge reservoir for scientists working in the field of Game Meat production chain.
